# Health ambassadors in the workplace: a health promotion intervention mobilizing middle managers and RE-AIM evaluation of outcomes

**DOI:** 10.1186/s12889-021-11609-8

**Published:** 2021-08-23

**Authors:** Keren L. Greenberg, Milka Donchin, Elisheva Leiter, Donna R. Zwas

**Affiliations:** 1 The Linda Joy Pollin Cardiovascular Wellness Center for Women, Hadassah University Medical Center, P.O.B. 12000, 91120 Jerusalem, Israel; 2 Braun School of Public Health and Community Medicine, Hebrew University and Hadassah University Medical Center, Jerusalem, Israel

**Keywords:** Workplace health promotion, Healthy setting, RE-AIM framework

## Abstract

**Background:**

The workplace provides an ideal setting for health promotion, as adults spend most of their day at work. Middle managers hold a strategic position to lead workplace health promotion. This study evaluates the outcomes of an intensive intervention training middle managers to promote health in the workplace.

**Methods:**

A workshop was designed and conducted to train female middle management employees to construct, implement, and evaluate a health promotion program in their workplace. Semi-structured interviews were carried out post-intervention to assess workplace health promotion outcomes according to the RE-AIM framework, and identify variables contributing to success. Additionally, questionnaires were distributed pre and post-program assessing personal health and self-efficacy changes.

**Results:**

Eighteen participants from 13 government offices, who serve 19,560 employees, completed the training course. Nine workplaces had workplace health promotion programs in progress 12 months after the course had ended, of which 8 made health promotion changes in organizational policy. Workplace RE-AIM scores showed that 8 workplaces were high or partial performers, and 5 were low or non-performers. Factors that increased the likelihood of successful interventions included management support, steering committee, comprehensive programming, conducting a needs assessment and flexibility in program implementation in the presence of challenges. Post course, participants reported increased health knowledge related to workplace health promotion (*p* < 0.001), and increased health promotion self-efficacy (*p* < 0.05).

**Conclusions:**

Training and continued guidance of middle managers resulted in the design and successful implementation of workplace health promotion interventions. A RE-AIM based assessment was found to be an effective method for evaluating multi-content workplace health promotion programs.

Registered at ClinicalTrials.gov, https://www.clinicaltrials.gov, registration number:

NCT03295136, registration date: 24/09/2017.

**Supplementary Information:**

The online version contains supplementary material available at 10.1186/s12889-021-11609-8.

## Background

The Ottawa Charter for Health Promotion states that health promotion (HP) activities should take place within the settings of people’s everyday lives, where they learn, work, play, and love [1]. As adults spend most of their day at work, the workplace is an ideal setting for health promotion [2, 3]. Additionally, the workplace is well-suited to multi-level interventions involving policy, environmental, organizational and interpersonal interventions. More so, as the demands of the workplace increase, workers spend more time behind their desks, eat high-calorie low nutrient foods, exercise less, and experience more stress [3].

In 2007, the 60th World Health Assembly endorsed a Global Plan of Action on Workers’ Health for 2008–2017, urging WHO member states to devise national policies and plans for implementation [4]. These efforts have been fruitful, as many studies in the past two decades have documented the success of workplace health promotion (WHP) interventions [5–12]. These interventions have improved employees’ physical and mental health through adoption of healthier habits [5–10], and have improved work productivity and satisfaction [11], as well as workers’ sense of commitment and responsibility to the organization [12].

Many studies describe the critical role of upper management support in the success of health promotion programs [13–15]. That said, middle managers, defined as the management level positioned between upper management and first-level supervisors [16], may be the most strategically positioned for WHP, as they have the legitimacy and authority to lead both top down and bottom up processes [17–19]. Although evidence exists of the advantages in generating work environment change through middle managers [20], there is a paucity of research and outcomes data on using this platform to promote health in the workplace by training middle managers as health promoters. Our study evaluates outcomes of a training and mentoring intervention for middle managers as health promoters within their organization, and assesses which program components facilitate a successful and sustainable intervention. This intervention’s primary aim was to train middle managers to design, implement, and evaluate a health promotion intervention in their workplace.

## Methods

### Program overview

#### Development

The “Health Ambassadors in the Workplace” health promotion intervention was developed by a project team including health promotion experts from the Linda Joy Pollin Cardiovascular Wellness Center for Women, representatives from the Council of Working Women, and experts in group facilitation. The project team included organizations focused on women’s health and empowerment, hence women were the target population for leading the WHP interventions. Additionally, most HR managers targeted in this intervention were women.

The intervention’s primary aim was to train female middle management employees to design, implement, and evaluate a health promotion intervention in their workplace, resulting in WHP in multiple organizations. The secondary aim was to increase health promotion self-efficacy and promote personal health-related change among the participants, in order to enable the implementation of WHP. Studies show that lay health promoters who have received training to promote personal and organizational health changes have proven to be more empowered and successful health promoters [18, 21].

In 2017, a pilot workshop was carried out and evaluated with 27 participants from nine workplaces. Feedback and insights gained from pilot outcomes were incorporated into the present intervention design, which took place during 2018–2019. Changes included the recruitment of middle-managers in positions enabling them to lead WHP, and augmented focus on personal health changes that increase mindedness to health promotion and motivate participants to be change agents.

#### Structure and content

The training course consisted of 16 sessions - 12 consecutive weekly sessions of 5 h, and 4 maintenance sessions of 4 h every 2 to 3 months during the following 9 months. Telephone-based mentoring sessions were then conducted every 4 months during an additional 12 months.

The curriculum was developed to enable the design of WHP programs according to the principles of the socio-ecological model [22]. Participants were trained to create programs targeting multiple levels of influence, including interpersonal, organizational, and policy. The curriculum structure also supported several practices associated with effective workplace interventions, including needs-assessment, tailoring of health messages, targeting multiple risk factors, and offering a variety of engagement methodologies [23]. The detailed course curriculum is presented in Supplement 1.

The 12-week intensive intervention was composed of two main stages: the first stage was dedicated to increasing health knowledge, enabling personal health behaviour change and promotion of personal health self-efficacy. The participants received lectures on health topics related to health promotion, including the Mediterranean diet and food label reading, physical activity (PA), smoking cessation, women’s health and stress management. Throughout the sessions, participants acquired personal change tools (e.g. overcoming barriers and set-backs, handling procrastination, personal empowerment exercises, etc.), set individual goals, participated in hands-on activities to enhance healthy skills and habits (e.g. healthy cooking competition, walking meetings, stress reduction exercises, physical activity exercises using office chairs, etc.), shared success stories and challenges, participated in women empowerment activities, and received group support. The second stage focused on the skills of WHP program design, including needs assessment, program development and implementation, and leadership and evaluation skills. Additional content included the principles of sustainable health promotion, social marketing, organizational development, and methods to foster personal and community change. Skills taught included preparing program budget, recruiting partners and fostering internal commitment. The course offered interactive workshops, close professional guidance and team building exercises, in order to create a health promoters network. The participants were encouraged to nominate a steering committee in their workplace and to target different population sectors. Additionally, they were required to present a WHP program to their employers by the end of the first 12 sessions and obtain their approval before implementation. Public health and organizational development professionals guided the process of designing the WHP programs and their details.

During the 12 months following the training course, mentoring sessions were conducted by phone every 4 months. The last phone mentoring session took place 1 year after the training course ended, 2 years after its initiation. During phone sessions, participants were asked about their progress, adherence to their original project plan, challenges or barriers, their WHP plans for the following months, and feedback from employees. Participants were also asked about process evaluation implementation and its results, based on their original evaluation plan. Guidance and professional support was offered when needed, as well as positive reinforcement.

#### Participant recruitment

This intervention targeted female Human Resource (HR) and employee welfare managers specifically, as these middle-management staff are ideally situated to make HP changes in their workplace [17–19]. They benefit from upper management support, have experience and expertise in leading programs, and the mandate to promote HP oriented processes as part of their job responsibilities.

Researchers and stakeholders contacted the Civil Service Commission (CSC) and recruited them to take part in the program. With the CSC’s approval and support, a call for proposal was sent out to all governmental offices. Those interested applied to participate in the program.

Eligibility for program acceptance necessitated commitment from senior management of the following: sending staff members to attend the training sessions, supporting and enabling the health promotion process in their workplace, and providing financial support for health promotion programming. All organizations, including those with pre-existing WHP programs, were eligible for inclusion in the intervention. Twenty-two participants from 13 governmental offices registered. Two of these government offices reported currently having WHP programs.

### Assessment

Baseline questionnaires assessed participants’ nutrition behaviours and weekly minutes of PA, knowledge of cardiovascular risk factors and symptoms, and self-efficacy as health promoters. The questionnaire was re-administered at the final session of the course.

Nutrition behaviors included adherence to the Mediterranean diet, which was assessed using a culturally-adapted and translated measure from the ATTICA study [24]. Western diet behaviors were assessed using selected questions from additional nutrition scales [25, 26]. PA was assessed using the Healthy Heart Score questionnaire, [25] and scored based on intensity level [27]. Self-efficacy as health promoters was measured using a 12 item health promotion leadership self-efficacy measure, adapted from a scale developed by the Myers-JDC-Brookdale Institute [28, 29] .

Post intervention evaluation also included an interview assessment, conducted as part of the mentoring phone calls every 4 months following the course, for 1 year. These semi-structured interviews were recorded and designed to assess planning, implementation and assessment of the WHP interventions. Interviews included 26 questions based on the criteria of the five RE-AIM dimensions [30]: Reach, Effectiveness, Adoption, Implementation and Maintenance (Table 1). This model conceptualizes the public health impact of an intervention and the total score represents intervention quality. The questions were created and tailored to both the program design and the expected outcomes. Questions addressing aims, objectives and evaluation were taken from the EQUIHP evaluation tool, a questionnaire facilitating the assessment and improvement of quality in health promotion programs [31].

**Table 1:** RE-AIM Dimensions for Workplace Interventions

Dimension	
Reach	‘Reach’ represents the number of individuals that were exposed to and affected by the program. The measure includes the percent of employees that were exposed to HP content, including via organizational digital media, and the percent of employees that participated in HP activities.
Effectiveness	‘Effectiveness’ represents the potential impact of the program as a result of adequate pre-planning, including needs assessment, clear definition of aims and goals and planned process evaluation.
Adoption	‘Adoption’ refers to the extent of which an organization adopted the program. It includes the establishment of a steering committee, integrating the program into the yearly work plan and budgeting it, and the involvement of both management and employees.
Implementation	‘Implementation’ refers to the consistency of delivery of the program as planned, the program’s scope and whether those implementing the program were from within the organization or outsourced.
Maintenance	‘Maintenance’ represents the sustainability of the program, including existence of policy changes and institutionalization of the health promotor role within the workplace, enabling the maintenance of WHP in the organization.

The interview assessment was completed by the interviewer through in-depth review of transcripts immediately following each interview. Twenty-three questions had a three-point scale: ‘yes’, ‘no’, or ‘partially’ (coded on a scale of 0–2, as per the EQUIHP scale). Three of the questions were nominal, and were categorized into three levels to allow for coding similar to the other 23 questions.

A second researcher independently listened to the interview recordings and scored the RE-AIM questions accordingly. The questions that were coded differently between the two researchers were marked, and after discussion between the researchers, a consensus score was determined.

Questions that were not applicable (e.g. asking about steering committee members when there was no committee) were coded as missing. An average of the RE-AIM scores was calculated per workplace, to determine level of performance.

For this analysis, success indicators were defined as participation of at least 50% of employees in a health behavior change intervention, implementation of a sustainable WHP program, modification of organizational policy to include WHP, and execution of the WHP program according to the original plan.

### Program documentation

During each interview, program graduates were asked to supply evidence of their work, including newsletters that were distributed, pictures of health promotion initiatives and activities, logos, yearly work plan, budgeting, etc. Assessment of documentation was included in the program evaluation, strengthening and validating the RE-AIM interview assessment.

### Data analysis

Data was analyzed using SPSS version 25. Frequencies were calculated to describe demographic variables. Paired t-tests were conducted to compare matched data of program participates in the evaluation of the training course. Fisher exact test was used to test associations between specific RE-AIM interview assessment variables and success indicators.

All methods were carried out in accordance with relevant guidelines and regulations.

## Results

Twenty-two participants were recruited from 13 government offices, who serve a total of 19,560 employees. The number of employees ranged from less than 500 to over 4000, with nearly half (46%) of the workplaces having between 500 and 2000 employees. Eighteen participants (82%) completed the course, attending at least 80% of the sessions. Four participants dropped out due to health reasons or organizational changes. One participant completed the course but did not plan a program due to organizational changes and lack of job security in her current position. The remaining participants designed a detailed WHP program, based on the needs assessment they performed. The programs included a written justification, clear SMART objectives, a budget, partner recruitment plan, a Gantt chart, a ‘plan B’ program, a marketing program aimed at upper management and employees, and an evaluation plan, which included process as well as outcome evaluations.

### Workplace outcomes

Four workplaces exposed at least 50% of their employees to health information aimed to raise awareness; total exposure in all 13 workplaces was approximately 4490 employees. In five workplaces, at least 50% of employees participated in an intervention targeting health behavior change; total participation in interventions in all 13 workplaces was approximately 4520 employees.

Only four out of the 13 workplaces that participated in the program had no active HP initiative. Examples of health topics and interventions appear in Table 2.

**Table 2:** WHP Intervention Topics

Topic	Intervention	Workplace ID
**Nutrition**	1. Healthier refreshment options at meetings/events/coffee corner	1, 3, 4,5, 6, 7, 8, 13
	2. Nutrition/healthy cooking workshops for employees	1, 3, 5, 7, 8, 13
	3. Nutrition lectures	3, 4, 5, 13
	4. Menu modification in food courts under nutritionist’s recommendation	1, 5, 8
	5. Change of organization food supplier to healthier one	8, 13
**PA**	1. Competitive sport groups	5, 8
	2. PA classes	1, 3, 4, 5, 7, 13
	3. PA breaks during work hours	1
	4. Running groups	5, 13
	5. Organization-wide sports day	1, 3, 4, 5, 7
	6. Subsidy of gym/pool membership	5
	7. Encouragement to take stairs or reduce sedentary time	4, 6
**Stress reduction**	1. Stress reduction workshops	1, 7, 5
	2. Yoga and meditation workshops	7, 13
**Screening tests**	1. Employee screening for high blood pressure and blood sugar by a nurse	1
	2. Employees encouragement to get recommended screenings/Subsidization of screenings	1, 8
**Smoking cessation**	1. Smoking cessation groups	1, 5, 7, 8
**Raising health awareness**	1. Health lectures	1, 3, 4, 5, 10
	2. Dissemination of health information and tips via organizational digital platforms	3, 4, 7
	3. Creation and distribution of paraphernalia with health content (calendars, holiday presents, pamphlets)	1, 7, 10
	4. Distribution of “healthy gifts”, such as branded water bottles, stress balls, resistance bands	4, 5, 7, 8
**Other**	1. Training of additional workplace health promoters	7, 13
	2. First aid courses	4, 7, 13
	3. Healthy shopping and budgeting workshops	13
	4. Cancer awareness lectures	7, 8
	5. Ergonomics workshop	4, 5, 7

Workplace RE-AIM scores are presented in Table 3. Workplace scores ranged from zero to a maximal 2; 6 workplaces were high performers (1.33–2), 2 were partial performers (0.66–1.33) and 5 were low or non-performers (0–0.66). Of the 13 workplaces, nine had HP programs in progress 12 months after the course had ended. Eight workplaces made health-promoting changes in organizational policy. Six workplaces officially appointed a health promoter in their organization (Table 4).

**Table 3:** Workplace Program RE-AIM Assessment Scores

Workplace ID	Num. of Employees	Employee Reach, HP Content	Employee Reach, HP Activities	Mean Effectiveness	Mean Adoption	Mean Implementation	Mean Maintenance	RE-AIM Avg.
**1**	500	1.00	2.00	1.83	2.00	1.71	1.25	1.63
**2**^**a**^	2500	0	0	–	0.50	0	0	0.10
**3**	550	2.00	1.0 0	1.00	1.50	1.17	2.00	1.45
**4**	160	2.00	1.00	1.00	1.50	1.33	2.00	1.47
**5**	1000	1.00	1.00	0.83	0.75	1.43	1.00	1.00
**6**	240	1.00	2.00	1.00	0.25	0.33	1.25	0.97
**7**	1200	2.00	2.00	2.00	2.00	2.00	2.00	2.00
**8**	650	1.00	2.00	1.67	2.00	1.86	2.00	1.76
**9**^**a**^	60	0	0	–	0	0	0	0
**10**	4000	1.00	0	0.67	1.00	0.33	0	0.50
**11**^**a**^	4200	0	0	–	0	0	0	0
**12**^**a**^	500	0	0	–	0.25	0	0	0.05
**13**	4000	2.00	2.00	1.33	0.75	1.71	1.50	1.55

Scores range 0–2, 0 = no implementation, 1 = partial implementation, 2 = full implementation

^a^Organization did not implement a program in the workplace, but may have had management support or an approved budget

**Table 4:** Percentage Rates of Workplaces that Achieved Positive Outcome According to Organizational Factors

WHP Factor (n)	Over 50% of employees participate in health activity	Program is sustainable	Changes were made in organization policy	Program was implemented according to plan
Steering Committee				
No (9)	11.1%	57.1%	44.4%	37.5%
Yes (4)	100.0%	100.0%	100.0%	100.0%
Sig.	0.007	0.212	0.098	0.255
Appointment of Health promoter in the organization				
No (7)	14.3%	40.0%	28.6%	50.0%
Yes (7)	66.7%	100.0%	100.0%	100.0%
Sig.	0.086	0.061	0.016	0.091
Approved budget				
No (5)	40.0%	50.0%	40.0%	40.0%
Yes (7)	42.9%	85.7%	85.7%	100.0%
Sig.	0.689	0.279	0.152	0.045
Management support				
No (2)	0%	0%	0%	0%
Yes (10)	50.0%	80.0%	80.0%	90.0%
Sig.	0.318	0.273	0.091	0.045
Flexibility in problem solving				
No (4)	0%	0%	0%	0%
Yes (4)	75.0%	100.0%	100.0%	100.0%
Sig.	0.071	0.067	0.014	0.029
Needs Assessment				
No (8)	12.5%	66.7%	50.0%	71.4%
Yes (5)	80.0%	80.0%	80.0%	80.0%
Sig.	0.032	0.576	0.315	0.636
Yearly work plan				
No (5)	20.0%	25.0%	20.0%	50.0%
Yes (8)	50.0%	100.0%	87.5%	87.5%
Sig.	0.315	0.024	0.032	0.236
Implemented by internal resources				
No (2)	50.0%	50.0%	50.0%	100.0%
Yes (7)	57.1%	100.0%	100.0%	100.0%
Sig.	0.722	0.222	0.222	–
Comprehensive program				
No (5)	20.0%	25.0%	20.0%	40.0%
Yes (7)	57.1%	100.0%	100.0%	100.0%
Sig.	0.247	0.024	0.010	0.045

Fisher’s Exact Test

This table reflects *N* = 13 workplaces. If the question did not apply to the workplace, it was considered missing data, for example, the number of respondents for outcomes related to program sustainability and implementation reflect only the workplaces that implemented WHP programs, and flexibility in problem solving only refers to those workplaces who reported significant challenges. All percentages were calculated with missing data excluded

### Predictors of successful outcomes

Table 4 examines the presence and absence of organizational factors and the percentages of workplaces that achieved positive outcomes. Of the four workplaces that did not implement health promotion programming, three lacked management support, and one had partial support. The number of employees reached was associated with the presence of a steering committee and the performance of a needs assessment. The four workplaces that had steering committees promoting the HP initiatives in their organizations addressed more health topics (4-5) than those without (0–3); the workplaces with steering committees were also more likely to create a logo and specific branding for their organization’s HP program.

Implementation of the programs according to plan was associated with flexibility in problem solving (self-reported) as well as having a comprehensive program, management support and budget allocation. The sustainability of the program was associated with incorporation of programming in to the yearly work-plan and with having a comprehensive program. Policy change was associated with flexibility, incorporation of programming in to the yearly work-plan, having a comprehensive program, and appointment of a HP promotor in the organization.

### Participant outcomes

All participants were female, with an average age of 49.2 + 8.4 (33–65), and 95% had a BA or higher degree. All participants were HR managers or held similar positions.

Post course, participants reported increased health knowledge that would enable engagement in WHP (*p* < 0.001), and their health promotion self-efficacy scores increased (*p* < 0.05). When describing their personal health behaviours, they reported reduced consumption of sweets (*p* < 0.01), and decreased consumption of refined grains (p < 0.05), as seen in Table 5.

**Table 5:** Participant Outcomes Pre and Post 12-week Course

Variable	n	Pre	Post	Sig.
**Average consumption of:**				
Fruit and Vegetables (servings per day)	14	2.95	3.12	0.330
Sweet drinks (servings per day)	14	1.47	0.54	0.056
Sweets (servings per day)	13	1.52	0.48	0.009
Refined grains (servings per day)	13	1.45	0.66	0.036
Nuts (servings per week)	14	1.96	2.71	0.150
Fish (servings per week)	13	1.26	2.04	0.067
Fried food (servings per week)	14	1.28	0.78	0.790
**Moderate PA** (mean minutes per week)	14	90.00	111.43	0.110
**Vigorous PA** (mean minutes per week)	14	32.00	46.78	0.331
**Self-efficacy as health promoters** (mean scale of 1–5)	14	3.12	3.38	0.030
**Health knowledge enabling WHP**	14	2.93	4.07	< 0.001

The data presented in this table were obtained from t-tests

## Discussion

This training and mentoring intervention mobilized middle managers to self-initiate, design and implement multi-component WHP initiatives, leading to implementation of WHP programs in 9 out of 13 workplaces. Additionally, it utilized the RE-AIM framework for a multi-component, multi-program evaluation. This study suggests that intensive WHP training of middle managers increased their engagement in WHP and led to programs that were both sustainable and led to health-promoting policy changes in the organization. This intervention led to WHP programs that exposed employees in 13 workplaces to health information aimed to raise awareness, and enabled approximately 4500 employees to actively participate in health promoting activities or to be exposed to health promoting policy changes.

### Mobilizing middle management

Middle managers have been identified as integral to shaping work environments that support behavior changes [20, 32], and play a key role in successful implementation of WHP [33]. A study of middle managers from 701 high-tech organizations in the UK [19] demonstrated that when these key personnel participate in decision-making within their organization, it leads to an increase in their strategy commitment (the extent to which one comprehends and supports the organizational strategy). That being said, a 2017 Danish study examined the role of middle managers in implementing WHP programs in six organizations and found that they feel uncertain about their role [33]. Exploratory interviews indicated that implementing WHP as a health strategy was a new discipline for them, and they needed more knowledge and tools to fulfill this new role. While the Danish study provides evidence of the benefits involved in including middle management in change processes in the workplace, it also emphasized the need for relevant training, educating, and empowerment, both in terms of health information and for leading processes of change. In general, trained and engaged lay health promoters prove to be more empowered and successful at promoting health compared with untrained health promoters [21]. Given their strategic placement in workplaces and training needs, the current intervention’s intensive WHP training and continued supervision of middle managers was necessary to reach WHP outcomes.

Personal health behavior performance appears to increase one’s likelihood of engaging in health promotion [34]. Given this relationship, our WHP training included a personal health behavior change component for middle managers. While this component likely improved their WHP engagement, the small numbers of participants in this study precluded determination of correlation between personal change and health promotion efficacy.

### Staff-initiated WHP

While standard WHP interventions may utilize external or commercially-developed programs [5–8], this intervention mobilized middle managers to initiate all steps of WHP (from conceptualization to implementation and evaluation), giving them more control and potentially increasing their investment and likelihood of implementation, which may improve sustainability. Studies show that involvement in the design and ownership of a HP program is vital to the a success of sustainable programs [35, 36]. Additionally, this method allows WHP programs to be tailored to each organization’s specific needs, culture, setting, and personnel, resulting in a variety of programs with different foci in each organization.

### Group-based intervention

The group setting enabled a single health promotion expert to efficiently train multiple managers simultaneously, both in terms of health knowledge and in the principles and practices of evidence-based health promotion. At the same time, mentoring sessions and follow-up individualized guidance was provided to each workplace. The group itself served as a support mechanism and forum for problem-solving and sharing of resources, which continued during the follow-up period. The group framework allows for cost-effective scaling up under real-world conditions, potentially allowing for greater reach while maintaining efficacy. Factors that increase the likelihood of the scalability of this intervention also include simplicity, capacity building, cost-effectiveness, promotion of evidence-based practices, engagement of local implementers and stakeholders, and engagement and activation of the health workers by promoting personal change [37, 38].

### RE-AIM assessment

The RE-AIM model is a known and commonly used framework to assess HP interventions [39]. It has been used to assess WHP initiatives as well, primarily for interventions focusing on a single topic, such as obesity prevention [40], walking programs [41], or PA programs [42]. In this study, the RE-AIM framework was adapted to be used to evaluate multiple-component interventions focusing on a variety of topics as well as to identify variables that lead to success.

### Program characteristics correlated with WHP success

This study suggests that upper management support was integral to successful implementation of HP programming, and the sites without management support were unable to proceed. These findings correspond with a study evaluating best practices in workplace health promotion that reviewed wellness programs in 812 organizations, and identified organizational and leadership support practices as essential factors to participation as well as health and medical cost impact [43]. Similarly, a recent study found that workers are more likely to both participate in WHP and engage in recommended health behaviors when they feel that their employers care about their health [44]. The study further asserts that when companies have a salaried HP coordinator or dedicated HP funding, employees perceive that the organization supports their health, motivating their WHP participation and behavior change.

Beer-Bost et al. drew similar conclusions regarding management support in their 2019 study [45], stating that the acceptance, effectiveness and maintenance of an HP project, especially WHP interventions, depend on strong employer support. Other factors found to be predictive in our study included budget approval, a yearly organizational HP program, and appointment of an organizational health promoter. All of these may be interpreted as concrete markers of upper management support, organizational commitment and resource allocation. While the literature points to the importance of upper management support for successful WHP [13–15], our study emphasizes this need, indicating that beyond mobilizing middle managers for WPH, implementation necessitates upper management support. It is important to note that, while all workplaces were required to provide a written and financial commitment on the part of upper management to WHP, as well as authorizing the time commitment of the participants prior to participation in the training, three of the workplaces did not ultimately manifest upper management support. Of these three, one workplace underwent major organizational change resulting in middle managers’ uncertainty in their positions, one changed their upper management mid-year, and the third would not financially support a WHP. In this study, personalized contact with upper management explaining the process, outcomes and expectations of the program seemed to improve their engagement. In addition, inclusion of upper management in the steering committee and presence at program presentations likely increased their involvement.

A key factor associated with successful implementation included the formation and activity of a steering committee. Steering committees are considered important to customization and enhancement of HP activities in the workplace [46], although limited data exist as to the effectiveness of these committees. A study of 23 worksites found that improvements made to the effectiveness of the steering committee in the second stage of the study through enhanced facilitator involvement and better definition of HP options through “how-to” documents led to modest improvements in employee nutrition and smoking behaviours [47]. Although few studies directly assess the role of steering committees, many observational studies cite benefits from functions performed by the steering committee, including employee involvement, communication at all levels of the organization, and measurement of outcomes [48]. This study provides quantitative observational evidence of enhanced outcomes in the setting of a steering committee.

The WHO’s 2009 “What Works” meta-analysis shows that multi-component interventions adapted to the local context are most successful [49]. Our study similarly found that both needs assessments that facilitate adaptation to organizational context and multi-component comprehensive programs were correlated with success indicators.

Flexibility in problem solving was also associated with WHP success indicators. A meta-analysis examining fidelity versus flexibility in implementing ten health promotion interventions showed that all interventions required changes and adaptions, such as recruitment methods, time allocation, budget adaptions etc. [50]. The interventions were evaluated using the RE-AIM framework, which identified intervention change (in the face of unexpected obstacles) as an important theme. While fidelity is important when implementing a pre-planned HP intervention, it is also crucial to be able to adapt and adjust the program to changing circumstances [50], whether it be lack of budget, support, or a shift in organizational culture.

### Limitations

This study is limited by the participation of women only, as these findings cannot be generalized to male middle managers, and by its length- this study took place over 2 years, too short a time to properly examine intervention long-term sustainability and impact on employee health outcomes. Additionally, since this study focused on WHP in government offices only, its findings may not be easily generalizable to the private sector.

Data collection was based on HR managers’ self-report of program impact, which may contain bias. The varied outcomes, however, suggest that findings reflected the experience within the workplace. The limited number of middle-manager participants and lack of variability in their health behavior change (almost all reported positive change) did not allow for exploration of the relationship between personal change and WHP success measures. The small sample size may have led to an inability to detect additional significant correlations. Future studies should include a larger number of workplaces examined for longer period of time, as well as additional quantitative and objective data, such as employee health outcomes.

## Conclusion

In this study, training and continuing guidance of middle managers resulted in the design and successfully implementation of WHP interventions in 9 out of 13 workplaces 1 year after course completion. Six of these workplaces scored high on the RE-AIM scale, suggesting that these interventions were likely to be effective. Factors that increased the likelihood of successful interventions included management support, steering committee, comprehensive programming, conducting a needs assessment and flexibility in program implementation in the presence of challenges. A RE-AIM based assessment was found to be an effective method for evaluating multi-content WHP programs.

## Supplementary Information

**Additional file 1: Supplement 1.** Health Ambassadors in the Workplace- Curriculum.

## Data Availability

The dataset used and analyzed during the current study are available from the corresponding author on reasonable request.

## Abbreviations

HP: Health promotion
WHP: Workplace health promotion
PA: Physical activity
HR: Human Resources
CSC: Civil Service Commission
RE-AIM: Reach, Effectiveness, Adoption, Implementation, Maintenance
