# Supplementary material for: Health ambassadors in the workplace: a health promotion intervention mobilizing middle managers and RE-AIM evaluation of outcomes
Source: BMC Public Health. 2021 Aug 23;21:1585. doi: 10.1186/s12889-021-11609-8 (PMC8383401; doi:10.1186/s12889-021-11609-8)
Supplement: Supplementary file 1 — Additional file 1: Supplement 1. Health Ambassadors in the Workplace- Curriculum. [file 12889_2021_11609_MOESM1_ESM.docx]

**Supplement 1.**

**Health Ambassadors in the Workplace- Curriculum**

| Month | Session | Content |
| --- | --- | --- |
| Month 1 | Session 1  (5 hours) | - Baseline Questionnaires - Lecture: Introduction to health promotion - Workshop: Introduction to creating change in health behaviors |
|  | Session 2  (5 hours) | - Lecture : Heart disease in women and the importance of a healthy lifestyle* - Lecture: Healthy nutrition* - Workshop: Basic principles of creating personal change |
|  | Session 3  (5 hours) | - Lecture: Physical activity* - Lecture: Prevention and cessation of smoking* - Workshop: Goals, barriers and resources for creating change, both personal and organizational |
|  | Session 4  (5 hours) | - Lecture/workshop: Dealing/handling stress and burnout, practice of Tai Chi Kung and additional stress relief methods* - Workshop: workplace assessment –mapping health promoting characteristics of the workplace (including resources, barriers, opportunities), methods of conducting needs assessment - Homework: Needs assessment in the workplace |
| Month 2 | Session 5  (5 hours) | - Lecture: Healthy nutrition part 2* - Workshop: beginning the design of programs-   - Defining goals based on needs assessments   - Creative brainstorming process for initial selection of projects - Division into work groups - Lecture: Organizational consultant- planning and creating a work plan, budget and handling opposition |
|  | Session 6  (5 hours) | - Panel - Learning from Success: health promoting workplaces, learning from peers' successful health promotion projects - Workshop: Promoting project-design in workgroups |
|  | Session 7  (5 hours) | - Workshop: Healthy cooking competition - Workshop: Leading the project in the organization   - organizational resources / internal organizational recruitment   - Importance of steering committee   - How to present the program creatively, recruiting employers and employees   - Brief initial presentation of project to the group and peer feedback |
|  | Session 8  (5 hours) | - Lecture: Social marketing and self-branding of the project and project leaders - Workshop: Leading the project in the organization   - Marketing the project in the internal organizational arena   - Creating budget and Gantt chart |
| Month 3 | Session 9  (5 hours) | - Workshop: Networking - the benefits of working together, finding channels for conserving the power group created in the course   - Continued work on project with individual guidance from staff |
|  | Session 10  (5 hours) | - Lecture: Time management, prioritization and coping with procrastination - Workshop: Principles of creating an engaging presentation - Workshop: Promoting projects, creating presentation for final meeting |
|  | Session 11  (5 hours) | Pre-summary meeting   - Internal presentation of all program-presentations, peer feedback - Workshop: Maintaining change |
|  | Session 12  (5 hours) | Presentation of programs to employers and other guests:   - Personal summary of participants - Presenting programs - Employer feedback - Distribution of certificates - Post survey |
|  | **Maintenance sessions** | Maintenance meetings are hosted in different workplaces who present their programs |
| Month 5 | Maintenance 1  ( 4 hours) | Lecture: topic decided by group (Menopause health effects)  Updates from participants  Workshop: Dealing with organizational politics |
| Month 7 | Maintenance 2  ( 4 hours) | Lecture: topic decided by group (Reading food labels)  Updates from participants  Workshop: Dealing with people opposed to change |
| Moth 10 | Maintenance 3  ( 4 hours) | Lecture: topic decided by group (Diabetes)  Updates from participants  Workshop: Creativity and flexibility in program implementation  Program accelerator |
| Month 12 | Maintenance 4  ( 4 hours) | Final meeting  Updates from participants, overcoming barriers  Workshop: Continuing the healthy-promoter-network  Planning ahead |

* After every professional lecture, group members discuss how to incorporate the health topic into organization/personal life.

© All rights reserved to the Linda Joy Pollin Cardiovascular Wellness Center for Women, Hadassah Medical Center, Jerusalem
